# Analytic Solution for a Complex Network of Chaotic Oscillators

**DOI:** 10.3390/e20060468

**Published:** 2018-06-16

**Authors:** Jonathan N. Blakely, Marko S. Milosavljevic, Ned J. Corron

**Affiliations:** Charles M. Bowden Laboratory, U. S. Army Aviation and Missile Research, Development, and Engineering Center, Redstone Arsenal, AL 35898, USA

**Keywords:** complex network, chaos, analytic solution, coupled oscillators

## Abstract

Chaotic evolution is generally too irregular to be captured in an analytic solution. Nonetheless, some dynamical systems do have such solutions enabling more rigorous analysis than can be achieved with numerical solutions. Here, we introduce a method of coupling solvable chaotic oscillators that maintains solvability. In fact, an analytic solution is given for an entire network of coupled oscillators. Importantly, a valid chaotic solution is shown even when the coupling topology is complex and the population of oscillators is heterogeneous. We provide a specific example of a solvable chaotic network with star topology and a hub that oscillates much faster than its leaves. We present analytic solutions as the coupling strength is varied showing states of varying degrees of global organization. The covariance of the network is derived explicity from the analytic solution characterizing the degree of synchronization across the network as the coupling strength varies. This example suggests that analytic solutions may constitute a new tool in the study of chaotic network dynamics generally.

## 1. Introduction

The unpredictable, random-like fluctuations of chaotic solutions can rarely be conveyed by a simple mathematical expression. However, recent studies have produced examples of chaotic analytic solutions to certain hybrid dynamical systems [1,2,3,4]. The discovery of these solutions was facilitated by the hybrid structure which separates the timing of the oscillation from the variation in amplitude. It might be expected that coupling between such dynamical systems would likely remove this analytical convenience and result in a larger system for which no analytic solution can be found. Here, we introduce a method for coupling solvable chaotic systems that preserves solvability. Notably, the method can accommodate a high degree of asymmetry in connection topology and parameter variation across the network. We describe a particular example in detail and show how the analytic solution facilitates an analysis of synchronization dynamics.

An individual isolated solvable chaotic system typically contains two parts: (1) a linear subsystem that evolves in the neighborhood of an unstable fixed point; and (2) a nonlinear feedback mechanism that occasionally switches the location of the fixed point. Many variations on this theme have been reported. The linear subsytem may be first, second, or, perhaps, even higher order [2,4,5]. The updates of the fixed point location bound the trajectory to a finite region in phase space. The set of possible locations for the fixed point may be finite, even binary, or infinite [2,6,7]. Different switching rules for the same set of fixed point locations can result in different attractor topologies [2,3]. The timing of the switching events can be regulated by the system itself or imposed externally [8,9]. However, in all cases, the linear nature of the evolution of all but a known countable set of switching times makes a concise statement of solutions possible.

Generally, any interaction between two chaotic systems that would each be solvable in isolation removes the required conditions for solvability. For example, if the timing of switching events is internally regulated, coupling can change the instantaneous frequencies of the oscillators in a complicated manner so that switching times are difficult to determine. However, we now introduce a form of coupling that is compatible with the solution approach that has been so successfully applied to isolated systems. The essential property of this coupling is that it only affects the location of each fixed point. It makes no other change to the linear evolution of the oscillators between switching events.

## 2. A Network of Coupled Oscillators

Consider a network of *N* oscillators, each with its own distinct parameters and phase. Let the evolution of the *n*th node satisfy the equation

(1) $${\ddot{u}}_{n}-2\beta_{n}{\dot{u}}_{n}+\left(\omega_{n}^{2}+\beta_{n}^{2}\right)\left(u_{n}-s_{n}\right)=0,$$

where $\beta_{n}$ and $\omega_{n}$ are constants defining the negative damping and natural frequency, respectively, of the node. The variable $u_{n}\left(t\right)\in ℜ$ oscillates harmonically about the fixed point $s_{n}\left(t\right)$ that changes value only at discrete times in which ${\dot{u}}_{n}=0$. Assuming the coupling to be defined later affects only the location of the fixed points, the form of Equation (1) ensures that these switching times are regularly spaced such that the *i*th such instant, $t_{n,i}$, satisfies

(2) $$t_{n,i}=\left(i-\phi_{n}/2\pi \right)\frac{T_{n}}{2},$$

where $\phi_{n}$ is the phase of oscillation at time t=0 and $T_{n}=2\pi /\omega_{n}$ is the period. At each $t_{n,i}$, the value of $s_{n}$ is updated according to the rule

(3) $$s_{n}\left(t_{n,i}\right)=\frac{e^{\beta_{n}\pi /\omega_{n}}u_{n}\left(t_{n,i}\right)+F_{n,i}}{1+e^{\beta_{n}\pi /\omega_{n}}},$$

where $F_{n,i}$ represents the influence of linked nodes. This value of $s_{n}$ is retained until $t_{n,i+1}$, i.e., the next time when ${\dot{u}}_{n}=0$. The coupling term $F_{n,i}$ is a function only of the states of the other nodes at their latest switching time. Specifically,

(4) $$F_{n,i}=\left(1-\epsilon \right)f_{n}\left(u_{n}\left(t_{n,i}\right)\right)+\frac{\epsilon }{k_{n}}\sum_{m=1}^{N}A_{nm}f_{m}\left(u_{m}\left(t_{m,\lfloor i\frac{T_{n}}{T_{m}}+\frac{\phi_{m}}{2\pi }-\frac{\phi_{n}T_{n}}{2\pi T_{m}}\rfloor }\right)\right),$$

where $f_{m}$ is a chaotic one dimensional map, $k_{n}$ is the degree of the *n*th node, ϵ is the coupling strength, and *A* is the adjacency matrix for the network. Since each node has its own frequency and initial phase, care must be taken to properly index the switching times. The floor function (indicated by the notation ⌊⌋) in the subscript on the update times is a concise notation for the accounting required to do just that. An analytic solution for the entire network beginning at time t=0 consists of expressions in terms of known functions for $u_{n}\left(t\right)$ and $s_{n}\left(t\right)$ satisfying Equation (1), consistent with initial data for $u_{n}\left(0\right)$, $s_{n}\left(0\right)$ and $\dot{u}\left(0\right)$, for every value of *n* from 1 to *N*. We now proceed to derive such an analytic solution.

## 3. Analytic Solution

Since the location of the fixed point, $s_{n}\left(t\right)$, is updated regularly at times $t_{n,i}$, it follows that

(5) $$s_{n}\left(t\right)=\sum_{i=0}^{\infty }s_{n}\left(t_{n,i}\right)\cdot r\left(\frac{t-t_{n,i}}{T_{n}/2}\right),$$

where r(t) is a unit square pulse. Without yet knowing the value of $s_{n}\left(t_{n,i}\right)$ for any *n* or *i*, we may formally treat Equation (1) as a linear system driven by a train of square pulses of various amplitudes determined by the value of $s_{n}\left(t_{n,i}\right)$ for each *i*. By the superposition principle, the response of the system to the train of pulses is equivalent to a sum of the responses to each pulse in isolation. Therefore, $u_{n}\left(t\right)$ must take the form

(6) $$u_{n}\left(t\right)=\sum_{i=-\infty }^{\infty }s_{n}\left(t_{n,i}\right)\cdot Q_{n}\left(t-t_{n,i}\right),$$

where

(7) $$Q_{n}\left(t\right)=\left\{\begin{array}{c} \left(1+e^{-\beta_{n}T_{n}/2}\right)e^{\beta_{n}t}\left(\cos\omega_{n}t-\frac{\beta_{n}}{\omega_{n}}\sin\omega_{n}t\right),\;t<0,\\ 1+e^{\beta_{n}\left(t-T_{n}/2\right)}\left(\cos\omega_{n}t-\frac{\beta_{n}}{\omega_{n}}\sin\omega_{n}t\right),\;0\leq t\leq \frac{T_{n}}{2},\\ 0,\;\frac{T_{n}}{2}\leq t \end{array}\right.$$

is the response of the *n*th node to a single square pulse of width $T_{n}/2$ [3]. Together, Equations (5) and (6) are analogous to the general solution of a linear ordinary differential equation in that they both contain a set of constants determined by the initial conditions. What remains in constructing an analytic solution for the entire network is to determine the value of $s_{n}\left(t_{n,i}\right)$ for all *n* and *i* consistent with the initial values $u_{n}\left(0\right)$, $s_{n}\left(0\right)$ and ${\dot{u}}_{n}\left(0\right)$ for all *n*.

To proceed, we observe that $u_{n}\left(t_{n,i+1}\right)$ can be related to $u_{n}\left(t_{n,i}\right)$ by the equation

(8) $$u_{n}\left(t_{n,i+1}\right)=-e^{\beta_{n}\pi /\omega_{n}}u_{n}\left(t_{n,i}\right)+\left(1+e^{\beta_{n}\pi /\omega_{n}}\right)s_{n}\left(t_{n,i}\right).$$

From this result, together with Equations (3) and (4), it follows that

(9) $$u_{n}\left(t_{n,i+1}\right)=\left(1-\epsilon \right)f_{n}\left(u_{n}\left(t_{n,i}\right)\right)+\frac{\epsilon }{k_{n}}\sum_{m=1}^{N}A_{nm}f_{m}\left(u_{m}\left(t_{m,\lfloor i\frac{T_{n}}{T_{m}}+\frac{\phi_{m}}{2\pi }-\frac{\phi_{n}T_{n}}{2\pi T_{m}}\rfloor }\right)\right).$$

Thus, the states of all nodes at switching times satisfy an asynchronously updated coupled map [10]. Equation (3) converts these coupled map iterates into the desired constants $s_{n}\left(t_{n,i}\right)$. With these constants in hand, the complete solution, Equations (5) and (6), can be constructed.

**Figure 1:**
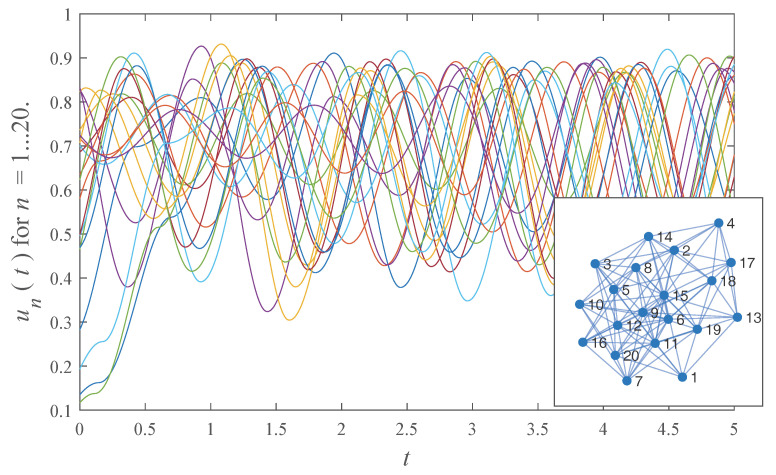
An analytic solution all 20 nodes of a heterogeneous random network. Each node is a hybrid dynamical system with a randomly chosen frequency, damping coefficient, and initial phase. Only the continuous states (i.e., $u_{n}\left(t\right)$ for n=1,2,…,20) of the hybrid systems are shown. The inset shows the randomly generated connection topology.

Equations (5) and (6) are valid for a large variety of models. The nodes of the network may have heterogeneous frequency and damping parameters, as well as initial phases. The topology of the network can have any structure that can be expressed by an adjacency matrix. For example, Figure 1 shows solutions for all nodes of a network containing 20 nodes with randomly distributed frequencies, damping constants, and relative phases. The connection topology, shown in the inset to Figure 1, was chosen randomly. The figure demonstrates how complex, high dimensional dynamics are amenable to analytic solution. To provide a more manageable illustration of the model, we now proceed to a simpler example of a network, its analytic solution, and further analysis of its synchronization properties enabled by this solution.

## 4. Example: A Star Network of Chaotic Oscillators

**Figure 2:**
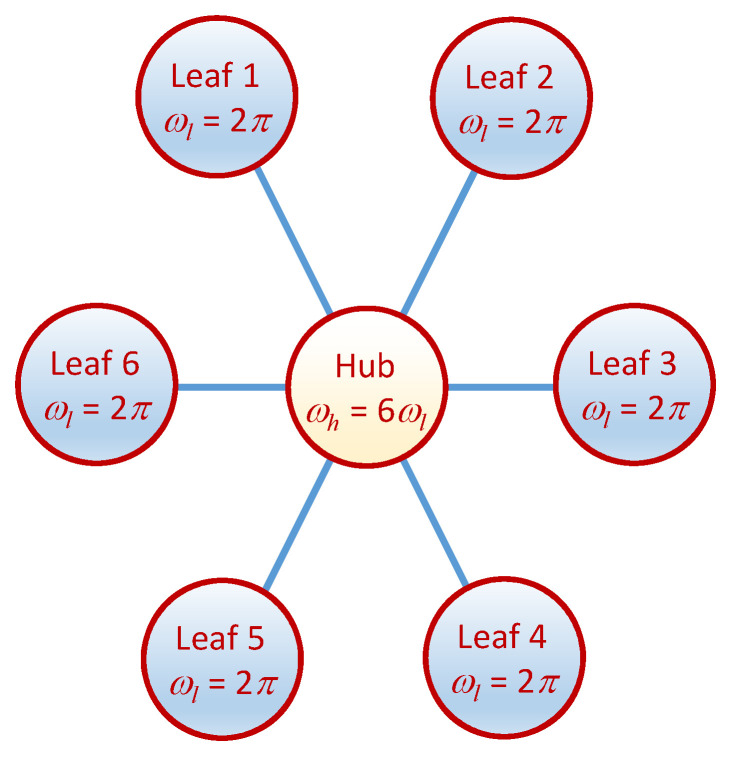
A network of hybrid oscillators with a star topology. The edges are bidirectional. The frequency of the hub is six times faster than that of the six leaves.

We now describe a particular example of a network of chaotic oscillators with an analytic solution in detail. For brevity, we choose not to present an example with the full complexity allowed for in the model of the preceding section. Thus, consider a small network with star topology as shown in Figure 2 whose updates occur in phase and are governed by a single chaotic map. The leaves of the network oscillate in phase with harmonic frequency $\omega_{l}=2\pi$ and the hub with a frequency $\omega_{h}=6\omega_{l}$. All nodes have the same damping coefficient β=1. The chaotic map appearing in the update rule of Equation (4) will take the form f(x)=rx(1−x), where r = 3.9.

The differential equations of the model are then

(10) $${\ddot{u}}_{h}-2{\dot{u}}_{h}+\left(\omega_{h}^{2}+1\right)\left(u_{h}-s_{h}\right)=0,$$

where $u_{h}\left(t\right)$ and $s_{h}\left(t\right)$ are the state variables of the hub, and

(11) $${\ddot{u}}_{n}-2{\dot{u}}_{n}+\left(\omega_{l}^{2}+1\right)\left(u_{n}-s_{n}\right)=0,$$

where n=1,2,…,6 and $u_{n}\left(t\right)$ and $s_{n}\left(t\right)$ are the state variables of the *n*th leaf. Here, we distinguish the equation of the hub notationally from those of the leaves to emphasize its unique role in the dynamics. Consequently, we introduce no adjacency matrix. Assuming the initial phase of the hub, $\phi_{h}$, and those of the leaves, $\phi_{n}$, are all zero, then, according to Equation (2), the *j*th update to the fixed point of the hub, $s_{h}$, occurs at time $t_{h,j}=j/12$, while those of the leaves occur at time $t_{l,j}=j/2$. At $t_{h,j}$, the value of $s_{h}$ is updated according to the rule

(12) $$s_{h}\left(j/12\right)=\frac{e^{\pi /\omega_{h}}u_{h}\left(j/12\right)+\left(1-\epsilon \right)f\left(u_{h}\left(j/12\right)\right)+\frac{\epsilon }{6}\sum_{m=1}^{6}f\left(u_{m}\left(\left\lfloor j/6\right\rfloor /2\right)\right)}{1+e^{\pi /\omega_{h}}}.$$

Likewise, at $t_{n,j}$, the value of $s_{n}$ is updated according to the rule

(13) $$s_{n}\left(j/2\right)=\frac{e^{\pi /\omega_{l}}u_{n}\left(j/2\right)+\left(1-\epsilon \right)f\left(u_{n}\left(j/2\right)\right)+\epsilon f\left(u_{h}\left(j/2\right)\right)}{1+e^{\pi /\omega_{l}}}.$$

The hub solution, $u_{h}\left(t\right)$, is of the form

(14) $$u_{h}\left(t\right)=\sum_{j=-\infty }^{\infty }s_{h}\left(j/12\right)\cdot Q_{h}\left(t-j/12\right),$$

and the leaf solutions, $u_{n}$ where n=1,2,…,6, are

(15) $$u_{n}\left(t\right)=\sum_{j=-\infty }^{\infty }s_{n}\left(j/2\right)\cdot Q_{l}\left(t-j/2\right),$$

where

(16) $$Q_{i}\left(t\right)=\left\{\begin{array}{c} \left(1+e^{-1/2}\right)e^{t}\left(\cos\omega_{i}t-\frac{1}{\omega_{i}}\sin\omega_{i}t\right),\;t<0,\\ 1+e^{\left(t-1/2\right)}\left(\cos\omega_{i}t-\frac{1}{\omega_{i}}\sin\omega_{i}t\right),\;0\leq t\leq \frac{\pi }{\omega_{i}},\\ 0,\;\frac{\pi }{\omega_{i}}\leq t, \end{array}\right.$$

with the subscript i=h for the hub and i=l for the leaves. The coefficients $s_{h}\left(j/12\right)$ weighting the basis functions of the hub solution are determined by iterating the equation

(17) $$u_{h}\left(\left(j+1\right)/12\right)=\left(1-\epsilon \right)f\left(u_{h}\left(j/12\right)\right)+\frac{\epsilon }{6}\sum_{n=1}^{6}f\left(u_{n}\left(\left\lfloor j/6\right\rfloor /2\right)\right).$$

The coefficients of the leaf solutions are generated by the equation

(18) $$u_{n}\left((j+1)/2\right)=\left(1-\epsilon \right)f\left(u_{n}\left(j/2\right)\right)+\epsilon f\left(u_{h}\left(j/2\right)\right),$$

where n=1,2,…,6.

**Figure 3:**
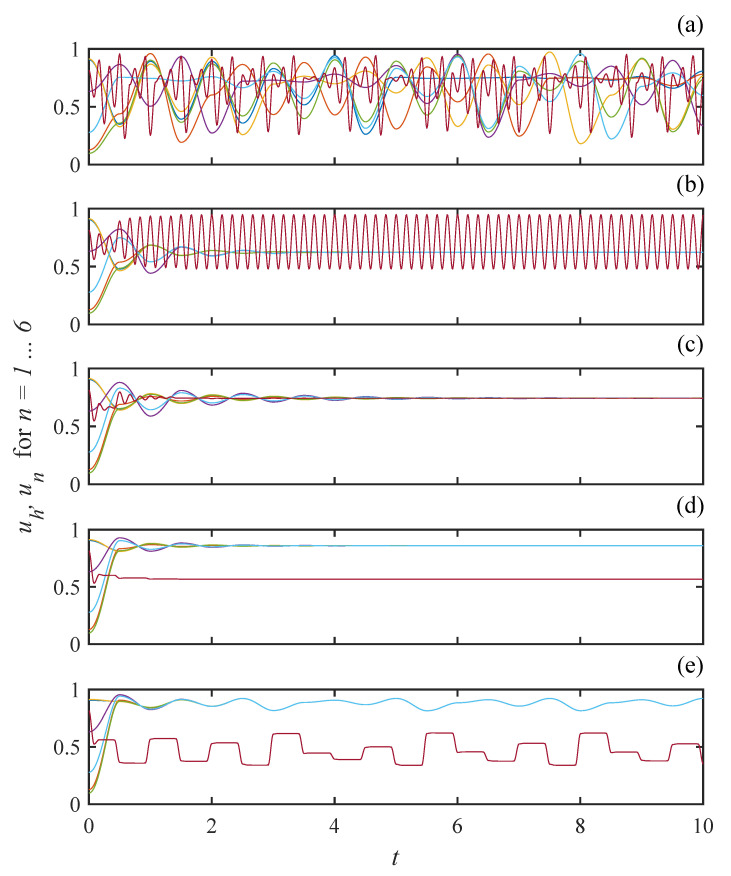
Analytic solutions for the star network as ϵ is varied. The red line shows the state of the hub. The coupling strength is (**a**) ϵ=0.1; (**b**) ϵ=0.4; (**c**) ϵ=0.6; (**d**) ϵ=0.8; and (**e**) ϵ=0.9.

Even though this example does not exhibit the full complexity allowed for in the model of Section 2, it still displays a rich variety of oscillatory states with non-trivial synchronization patterns. We now survey some of the observed behaviors as the coupling strength ϵ increases from zero. Exemplary time series are shown in Figure 3. Each waveform is a plot of the analytic solution, either Equations (14) or (15), using a randomly chosen initial condition, not a numerical solution of the dynamical system.

Figure 3a shows time series of each oscillator with ϵ=0.1, i.e., relatively weak coupling. The waveform of the hub (red line), $u_{h}\left(t\right)$, is easily identified by its fast irregular oscillations. Overall, there is little coherence apparent in the global state. However, close inspection reveals small sets of leaves do show strong temporary correlation. Thus, despite the weakness of the coupling and the fact that all interactions between leaves are mediated by the chaotic hub, the network displays a disposition towards temporary collective organization.

A strikingly different behavior is displayed in Figure 3b where the coupling strength is increased to ϵ=0.4. Here, the oscillations of the leaves have all given way to a steady state while the hub settles quickly into a nearly sinusoidal fast periodic orbit. To see why the oscillation of the hub does not disturb the steady state of the leaves, recall that in Equation (13) the dynamics of the leaves are only affected by the state of the hub at half integer values of *t*. At these times, $u_{h}$ is always at the maximum value of its periodic orbit. Thus, while the hub oscillates, the leaves are driven by a constant value. In the case where the steady state is taken as the initial condition, the iterates of Equation (18) produce a constant value of $s_{n}\left(t\right)$ for all *n* and *t*. In this case, it is straightforward to evaluate the infinite summation in Equation (15) to show it is constant all of the time.

Yet another organized global state is shown in Figure 3c where all nodes, hub and leaves settle to the same steady state value with ϵ=0.6. Equations (17) and (18) imply the steady state value of $u_{h}$ and $u_{n}$ for all *n* is 1−1/r. Accordingly, Equations (14) and (15) can be summed explicitly to show the steady state evolution of the continuous time network. The appearance of a homogeneous steady state in a network is sometimes referred to as amplitude death [11]. As ϵ is further increased, the steady state of the hub separates from that of the leaves as shown in Figure 3d where ϵ=0.8. This inhomogeneous steady state is an example of oscillator death [12]. A transition from amplitude to oscillator death was reported early on by Turing [13], but this phenomenon has received significant attention more recently [14,15,16,17].

Increasing ϵ even further to 0.9 brings about a revival of oscillation as shown in Figure 3e. Notably, the hub oscillates here at the slower frequency of the leaves. Both hub and leaves evolve aperiodically with complete synchronization between the leaves and some degree of coherence between the leaves and the hub.

The various time series in Figure 3 demonstrate the wide variety of behaviors displayed by this solvable network model. States with various degrees of collective coherence and transitions between them have been studied in other models using numerical simulations [12,14,15,18]. In the next section, we demonstrate the value of an analytic solution by providing an exact analysis of network dynamics without reliance on numerical methods.

## 5. Exact Expression for Covariance Matrix of the Star Network

Networks are typically high dimensional systems that can produce overwhelming quantities of time series data. Thus, it is often more practical to examine statistical properties rather than the details of the evolution of single nodes. Here, we give an example of how the solutions introduced in the previous section facilitate statistical studies of network dynamics. Specifically, we analyze synchronization among the leaves of the network in the range of weak coupling below the transition to amplitude death. We quantify the degree of synchronization of the network as a whole in terms of a normalized covariance matrix characterizing correlations between the six leaf nodes. With the analytic solution in hand, we can derive an exact expression for the elements of this matrix.

We define the normalized covariance *R* as a six by six matrix with elements

(19) $$R_{ij}=\lim_{T\to \infty }\frac{\int_{-T/2}^{T/2}u_{i}\left(t\right)u_{j}\left(t\right)dt}{\sqrt{\int_{-T/2}^{T/2}u_{i}^{2}dt}\sqrt{\int_{-T/2}^{T/2}u_{j}^{2}dt}},$$

where i,j=1,2,…,6. Each matrix element is a cross correlation between waveforms of solvable oscillators. Exact expressions for such correlations have been derived previously and we follow that approach here [19]. The structure of each waveform as an infinite sum of basis functions allows us to replace the limit as *T* goes to infinity with the limit as $N_{b}$, the number of basis functions, going to infinity giving

(20) $$R_{ij}=\lim_{N_{b}\to \infty }\frac{\sum_{m,n=-N_{b}}^{N_{b}}s_{i}\left(m\right)s_{j}\left(n\right)J\left(\frac{m}{2}-\frac{n}{2}\right)}{\sqrt{\sum_{m,n=-N_{b}}^{N_{b}}s_{i}\left(m\right)s_{i}\left(n\right)J\left(\frac{m}{2}-\frac{n}{2}\right)}\sqrt{\sum_{m,n=-N_{b}}^{N_{b}}s_{j}\left(m\right)s_{j}\left(n\right)J\left(\frac{m}{2}-\frac{n}{2}\right)}},$$

where

(21) $$J\left(n/2\right)=\int_{-\infty }^{\infty }Q_{l}\left(t\right)Q_{l}\left(t+n/2\right)dt$$

is the overlap integral for two instances of the basis pulse function $Q_{l}\left(t\right)$ displaced by a lag n/2. The overlap integral J(n/2) can be evaluated exactly [19,20] giving

(22) $$J\left(0\right)=\frac{\omega_{l}^{2}-1+\left(\omega_{l}^{2}-3\right)/\left(2\sqrt{e}\right)}{1+\omega_{l}^{2}},$$

while, for |n|>0,

(23) $$J\left(n/2\right)={\left(-1\right)}^{n}e^{-n/2}\left(\omega_{l}^{2}-3\right)\left[\frac{2+e^{1/2}+e^{-1/2}}{4(1+\omega_{l}^{2})}\right].$$

Thus, we now have exact expressions for the elements of the covariance matrix of a network of chaotic oscillators. A matrix element of value one indicates complete synchronization between the associated pair of leaves.

**Figure 4:**
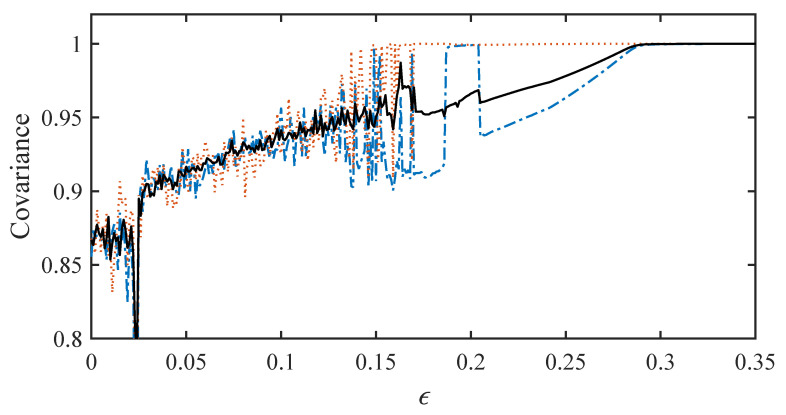
Elements of the covariance matrix as ϵ is varied. The average of the elements is shown in black, while $R_{2,5}$ and $R_{1,4}$ are shown in red and blue, respectively.

The covariance is plotted in Figure 4 using analytic solutions containing 195 basis functions. The solid black line is the average over all matrix elements. It indicates the global state of synchronization. The non-zero correlation at zero coupling is due to the fact that $u_{i}\left(t\right)$ has a non-zero mean. This effect accounts for the average covariance near 0.87 when ϵ=0. As ϵ increases, the average covariance increases until global synchronization occurs with ϵ=0.3. The synchronized state is periodic like the solutions shown in Figure 3b.

We may also examine individual matrix elements to observe localized synchronization at coupling strengths less than ϵ=0.3. The dotted red line in Figure 4 shows $R_{2,5}$, the correlation between leaves 2 and 5. This pair of leaves reaches synchronization around ϵ=0.15, a much weaker coupling strength than is required for global coherence. Even before this point, some values of coupling strength produce an $R_{2,5}$ near unity. A quite different behavior is displayed by oscillators 1 and 4. The dash-dotted blue line shows $R_{1,4}$. This pair shows sporadic coherence at weak coupling strengths. However, $R_{1,4}$ only approaches the average value very close to ϵ=0.3.

For brevity, we do not show the other matrix elements here, but each can be examined individually to fully characterize partial synchronization under weak coupling in this star network. Additionally, correlations with the hub oscillator could also be calculated analytically to reveal the transition at large coupling strengths to complete global coherence or even the state of synchronized chaos shown in Figure 3e.

## 6. Conclusions

In this paper, we have introduced a new tool for the study of dynamics on complex networks: the analytic solution of networks of chaotic hybrid oscillators. More specifically, we have shown a form of coupling between nodes that preserves the solvability of the nodes themselves. A detailed example of the coupling was described for a star network whose hub had a faster natural frequency than its leaves. Solutions corresponding to several examples of nontrivial network dynamics were presented including states of amplitude and oscillator death. The analytic solution was used to derive an exact expression for the covariance matrix characterizing correlations between the leaves.

The approach allows for many forms of network inhomogeneity. Here, we considered parameter variations, phase variations, and arbitrary coupling topologies. However, straightforward extensions of the model would easily incorporate even more diversity. For example, the nodes could be populated with solvable systems of different orders. In addition, the coupling could be made intermittent allowing for a time varying network. The main challenge in implementing these extensions is the bookkeeping involved when every node is relatively unique.

Finally, the usefulness of this new tool in network science will only be fully demonstrated when the model is used to reveal some new collective phenomenon or to bring light to some effect already under study. Thus, our current efforts are focused on applying the model to the study of topics of current interest in network science such as explosive transitions [21,22], symmetry breaking [18,23], and control [24,25].
